# Patients’ and Providers’ Perspectives on and Needs of Telemonitoring to Support Clinical Management and Self-care of People at High Risk for Preeclampsia: Qualitative Study

**DOI:** 10.2196/32545

**Published:** 2022-02-07

**Authors:** Maria Aquino, Janessa Griffith, Tessy Vattaparambil, Sarah Munce, Michelle Hladunewich, Emily Seto

**Affiliations:** 1 Institute of Health Policy, Management and Evaluation Dalla Lana School of Public Health University of Toronto Toronto, ON Canada; 2 Centre for Global eHealth Innovation Techna Institute University Health Network Toronto, ON Canada; 3 Institute of Medical Science University of Toronto Toronto, ON Canada; 4 Women’s College Hospital Institute for Health System Solutions and Virtual Care Women’s College Hospital Toronto, ON Canada; 5 Department of Occupational Sciences and Occupational Therapy University of Toronto Toronto, ON Canada; 6 KITE-Toronto Rehabilitation Institute University Health Network Toronto, ON Canada; 7 Division of Nephrology Department of Medicine Sunnybrook Health Sciences Centre Toronto, ON Canada

**Keywords:** high-risk pregnancy, blood pressure, preeclampsia, telemonitoring, home monitoring, mHealth

## Abstract

**Background:**

Preeclampsia is one of the leading causes of maternal mortality worldwide, with a global prevalence at 2%-8% of pregnancies. Patients at high risk for preeclampsia (PHRPE) have an increased risk of complications, such as fetal growth restriction, preterm delivery, abnormal clotting, and liver and kidney disease. Telemonitoring for PHRPE may allow for timelier diagnosis and enhanced management, which may improve maternal and perinatal outcomes.

**Objective:**

The objective of this study is to determine the perceptions and needs of PHRPE and their health care providers with respect to telemonitoring through semistructured interviews with both groups. This study explored (1) what the needs and challenges of monitoring PHRPE are during pregnancy and in the postpartum period and (2) what features are required in a telemonitoring program to support self-care and clinical management of PHRPE.

**Methods:**

This study used a qualitative descriptive approach, and thematic analysis was conducted. PHRPE and health care providers from a high-risk obstetrical clinic in a large academic hospital in Toronto, Canada, were asked to participate in individual semistructured interviews. Two researchers jointly developed a coding framework and separately coded each interview to ensure that the interviews were double-coded. The software program NVivo version 12 was used to help organize the codes.

**Results:**

In total, 7 PHRPE and 5 health care providers, which included a nurse practitioner and physicians, participated in the semistructured interviews. Using thematic analysis, perceptions on the benefits, barriers, and desired features were determined. Perceived benefits of telemonitoring for PHRPE included close monitoring of home blood pressure (BP) measurements and appropriate interventions for abnormal BP readings; the development of a tailored telemonitoring system for pregnant patients; and facilitation of self-management. Perceived barriers to telemonitoring for PHRPE included financial and personal barriers, as well as the potential for increased clinician workload. Desired features of a secure platform for PHRPE included the facilitation of self-management for patients and decision making for clinicians, as well as the inclusion of evidence-based action prompts.

**Conclusions:**

The perceptions of patients and providers on the use of telemonitoring for PHRPE support the need for a telemonitoring program for the management of PHRPE. Recommendations from this study include the specific features of a telemonitoring program for PHRPE, as well as the use of frameworks and design processes in the design and implementation of a telemonitoring program for PHRPE.

## Introduction

Preeclampsia is a progressive multisystem syndrome that involves new-onset hypertension and end-organ dysfunction or proteinuria in the second half of pregnancy or postpartum [1]. The prevalence of preeclampsia is 2%-8% worldwide [2]. The prevalence is higher in first-time pregnancies, in people of advanced maternal age, in people who have had preeclampsia in the past, and in those with preexisting medical conditions (eg, chronic hypertension, kidney disease, diabetes mellitus) [3]. People who are diagnosed with preeclampsia are at an increased risk of both obstetrical complications, such as fetal growth restriction and preterm delivery [4], as well as medical complications, such as abnormal clotting, peripheral and pulmonary edema, and liver and kidney dysfunction [5]. Additionally, preeclampsia increases the long-term risk of developing hypertension, ischemic heart disease, stroke, venous thromboembolism, kidney failure, and death [6-8]. Hypertensive disorders of pregnancy, including chronic hypertension, gestational hypertension, and preeclampsia [8], are a leading cause of maternal mortality worldwide [7]. Identifying patients at high risk for preeclampsia (PHRPE) may improve the probability of a timely diagnosis, which may enhance maternal and perinatal outcomes [9].

Home blood pressure (BP) monitoring may play a role as an adjunct to managing PHRPE for developing preeclampsia, because it allows for more frequent BP readings and may potentially lead to timelier detection of preeclampsia [10]. Home BP monitoring may also be beneficial in assessing the BP control of pregnant people with chronic hypertension [10]. The 2013 American College of Obstetricians and Gynecologists Guidelines recommend home BP monitoring for pregnant people with gestational hypertension, chronic hypertension, and uncontrolled BP [1].

Digital health interventions (DHIs), such as telemonitoring, can provide a personalized approach to the outpatient monitoring of PHRPE [11,12]. Specifically, telemonitoring enables health care providers to have access to real-time information about their patient’s home BP readings for clinical decision support, such as medication changes or delivery of the baby. Telemonitoring can also enable targeted and timely self-management instructions for PHRPE based on their current and trending BP readings. In our scoping review, published in 2020, we identified only 20 studies exploring telemonitoring of patients at high risk for hypertensive disorders of pregnancy [13]. These papers described telemonitoring interventions in countries such as the United Kingdom, the United States, and Belgium, but there were no Canadian studies published on telemonitoring interventions for patients at high risk for hypertensive disorders of pregnancy at the time [13]. Although we determined that telemonitoring could provide benefits for managing patients at high risk for hypertensive disorders, more research is needed to understand how to develop and implement such programs, as well as to prove its safety and effectiveness [13]. To the best of our knowledge, there has been no recent Canadian study published on the topic since our scoping review. Recently, 3 studies from the United Kingdom have explored the perspectives of women [14] and health care providers [15] as well as a design and implementation approach [16] for BP self-monitoring.

The overall objective of this study is to determine the perceptions and needs of PHRPE and their health care providers with respect to telemonitoring. There were 2 specific research questions for this study: (1) What are the needs and challenges of monitoring PHRPE during pregnancy or in the postpartum period? (2) What are the features required in a telemonitoring program to support self-care and clinical management of PHRPE?

## Methods

### Study Design

This study adopted a qualitative descriptive approach [17]. PHRPE and health care providers involved in their care from a high-risk obstetrical clinic at a large academic hospital in Toronto, Canada, participated in individual semistructured interviews. The study was approved by the research ethics boards of the University Health Network (#18-5535) and the Sunnybrook Health Sciences Centre (#295-2018). This research followed the guidance of the Consolidated Criteria for Reporting Qualitative Research [18].

### Participants and Recruitment

This study used purposive sampling with PHRPE and health care providers. PHRPE were eligible for the study if they were identified as being at high risk for preeclampsia, aged 18 years or older, able to communicate in English, pregnant or up to 6 weeks postpartum, and currently self-monitoring home BP measurements. Eligible patients were identified and were informed about the study by their physician during a clinic visit and were asked whether they would be willing to speak to the research coordinator (author MA) to participate in the study. The researchers had no prior relationship with the participants. The patients were also provided with a letter of invitation with further information that stated that their health providers would not know the identity of the study participants. Health care providers associated with the high-risk obstetrical clinic who were involved in the care of PHRPE were also invited to participate in a semistructured interview. They were invited to participate via email by the clinical site lead and could have various clinical roles, including general practitioners, obstetricians, nurse practitioners, nurses, and pharmacists. PHRPE and health care providers interested in participating were contacted by the research coordinator, who provided the details of the study and answered any questions of the potential participants prior to obtaining written consent. Recruitment continued until the data had reached saturation, which was anticipated to be between 5 and 15 participants in each of the 2 groups.

### Data Collection and Analysis

The interviews were conducted in person by the research coordinator (MA) in a private room at the clinic. The research coordinator had experience interviewing patient participants and had graduate-level training in research, as well as a background in nursing for the adult cardiac population. Each interview lasted between approximately 20 and 30 minutes. The interview guides are provided in Multimedia Appendix 1. The interviews were digitally recorded and transcribed verbatim by a professional transcriptionist for analysis. Repeat interviews did not occur.

Thematic analysis, as described by Braun and Clarke [19], was conducted. Two researchers (authors MA and JG, both she/her) coded several transcripts together to develop a common coding framework. Both researchers had advanced-level training for coding qualitative interviews. After the framework was established, the researchers separately coded each interview to ensure each interview was double-coded. The researchers discussed the codes to resolve any discrepancies. During this discussion, the codes were grouped into initial themes. The codes and themes were then refined through discussion by the larger research team. The software program NVivo version 12 (QSR International, Doncaster, Victoria, Australia) was used during the analysis of the transcripts to help organize the codes.

## Results

### Participant Demographics

In total, 7 patients participated in the study and ranged from being 12 weeks pregnant to 2 months postpartum. Many patient participants were referred to this clinic due to chronic kidney disease that caused hypertension during pregnancy. All patient participants measured their BP at home with varying degrees of frequency. The most common frequency of BP self-measurement was 3 times per day, with 3 (43%) of 7 patient participants using this schedule. Most patient participants also used a DHI before pregnancy to monitor other health conditions, such as diabetes, activity, and fertility. Only 1 (14%) of 7 patient participants used a mobile app, called QardioArm, to monitor their BP during pregnancy. Further details on the characteristics of the patient participants are shown in Table 1.

Most staff in the clinic also participated in the study, including specialists, staff physicians, and a nurse practitioner, with 4 (80%) of 5 providers being female. None of the providers had previously recommended the use of telemonitoring tracking technology for their patients. There were 5 provider participants, of whom 4 (80%) were female and 1 (20%) was male. The provider participants comprised 4 (80%) physicians and 1 (20%) nurse practitioner. In general, the health care providers were experienced in the fields of maternal-fetal medicine as well as nephrology.

**Table 1:** Demographics of patient participants.

Patient	Reason for referral to clinic	Frequency of BP^a^ self-measurements	Use of DHI^b^ before pregnancy	Use of DHI to monitor BP during pregnancy
1	Chronic kidney disease	3 times per day	No	No
2	Adrenal tumor, hypertension	6-10 times per day	Yes, for diabetes	No
3	Polycystic kidneys, hypertension	2 times per day	Yes, fertility tracker	Yes, QardioArm
4	Hypertension	3 times per day	Yes, step tracker	No
5	Nephrotic syndrome	3 times per day	No	No
6	Kidney disease	With symptoms	Yes, step tracker	No
7	Hypertension	2 times per day	Yes, Carrot app	No

^a^BP: blood pressure.

^b^DHI: digital health intervention.

### Themes From the Interviews

During the interviews, participants discussed how they envisioned a telemonitoring program could be used in the care of patients at the clinic. The following themes emerged within the categories of perceived benefits of, perceived barriers to, and desired features of telemonitoring for PHRPE.

#### Perceived Benefits of Telemonitoring for PHRPE

The perceived benefits were as follows:

- Telemonitoring PHRPE may provide close monitoring of home BP measurements and appropriate interventions for abnormal BP readings: Providers indicated that patients at risk for preeclampsia require close and frequent monitoring between clinic appointments. Some providers stated that telemonitoring could allow for remote observation of patients who have already been identified as having BP values that could change suddenly and unpredictably.

I think it can help some patients, yeah, I mean, the[se] patients [are] definitely one [of] the highest [risk] groups who really have to watch blood pressure quite carefully. Yeah, so this wouldn't be, it wouldn't probably [be], something I would use with the majority of patients who are at risk but in terms of blood pressure in such an intensive manner. We're talking about patients who already either have chronic hypertension that needs to be managed and followed closely or patients who are already diagnosed with mild preeclampsia and are being managed as outpatients. That probably would be the target population.

Provider 4

Additionally, telemonitoring may allow for improved access to perinatal health services for patients who may not have easy access to these resources.

They’re watched very carefully [in the] clinic, but there are women that are not watched carefully in other obstetrical population[s] . . . not everybody’s got that kind of resource; so then, you have to think out [if it could] be even a more useful tool for women to self-identify and come to triage maybe in time before they get too sick.

Provider 5

- Telemonitoring PHRPE may provide tailored information and interventions for pregnant patients: Providers reported that currently available telemonitoring systems are not tailored for pregnant people, which causes provider hesitancy in recommending them to patients.

So there hasn’t been [a telemonitoring system] that’s been sort of really tailored right for our population . . . they haven’t been sort of detailed enough for my pregnant women—so I think it could happen, but we don’t have the right one yet.

Provider 5

When patients were asked how they currently manage their BP between clinic visits, some patients mentioned that they resorted to using internet search engines for information regarding symptoms they were experiencing. Patients found information that was not specific to preeclampsia. A BP telemonitoring system that is tailored specifically to patients at risk for preeclampsia was thought to be useful.

It’s different criteria for different [patients]—like if you’re diabetic . . . and you’re a certain age or, you know, [there are] different criteria for different things . . . because what I find that even as a patient, you know, I find that even with a nursing background, sometimes I don’t know when to go to emergency, you know. I’m pregnant now, and I’m bleeding . . . should I go to [the emergency department] or should I not’, and I would imagine that’s the same thing that other people who don’t have a health care background may struggle with.

Patient 3

Patients expressed a desire to include contextual information related to their BP measurements (eg, medications taken, emotional and physical states, time of day) to provide their health care providers with more information about any potential influencing factors on the measurement.

I don't know if clinicians would want to know when you took a blood pressure medication, like did you take your blood pressure medication, like ’cause you don't [want] to assume that they did cause some people might not have. Or . . . if they want to have a comment section where you can write I've been feeling sick or I'm in a lot of pain or something like that. So people can write a comment next to the blood pressure results.

Patient 7

Providers noted that acquiring personal information about what patients are experiencing allows them to make better decisions on their care.

I think it’s crucial keeping a diary in pregnancy and for preeclampsia . . . [It allows me] to adjust medications and [know] how severe things are.

Provider 3

- Telemonitoring for PHRPE may facilitate self-management in patients: Self-management in this context can include actions taken, as needed, based on symptoms and self-monitoring of BP. An example of self-management would be a pregnant patient going to the emergency department for higher-than-normal BP readings and accompanying symptoms, such as a headache. Both clinicians and patients indicated that a telemonitoring system that educates patients on self-management practices could be empowering and could build their self-confidence in managing their condition. The study participants expressed that this sense of empowerment could help create positive habits for patients, including regular self-monitoring of their BP and an awareness of actions to take when the results are outside of the normal range.

You have to also make sure you’re empowering the patient to do self‑management, so if they are just thinking somebody else is watching . . . then that’s not actually self-management, so that’s the key, is to find an app that works but still encourages [patients] to take a very active part in their health care.

Provider 5

I think [an app] empowers people to be more proactive about their health and what they should do and who they should reach out to. People may take their [blood pressure] readings or may not and . . . they don’t know when to come in to see their family doctor or their specialists. It’s also a waste of resources for emergency teams or the emergency department . . . when you go in unnecessarily; so I think it can be useful.

Patient 3

#### Perceived Barriers to Telemonitoring PHRPE

The perceived barriers were as follows:

- Financial and other personal barriers to telemonitoring: Clinicians and patients highlighted the need to address financial barriers (eg, cost of a home BP monitor) to enhance patient BP self-monitoring.

Some people would not have the financial means to get a home blood pressure device, and going to [the] pharmacy, it's time demanding, it's complicated.

Provider 4

And if the [telemonitoring is] covered under insurance, [because] they can get pricey . . . especially if the doctor said you need to [monitor your blood pressure at home], if [providers] can write [a] prescription and the private insurance companies . . . can cover up to 70% [of the cost] or something like that.

Partner of patient 5

A telemonitoring system would need to demonstrate an added benefit or value in the patient’s care management, especially for those patients who currently manage their health without technology.

To be honest with you, it’s, I guess, it’s too much work, and I’m not really into, like, the whole technology thing.

Patient 5

I think the patient has to be convinced there is an issue, and I think sometimes the only way is if the patient is made to lead her condition and also to take charge. That’s the only way I feel she would take it seriously. And I think making them record or making them understand more will help motivate them. So I think if that does exist, that app, or whatever program, I think it would actually really help, because we do ask patients to record [blood pressure measurements]. I ask them to record.

Provider 3

Finally, access to the appropriate equipment, such as validated BP cuffs, for pregnant patients may pose as a barrier to the telemonitoring of this group.

Not all blood pressure cuffs are created equally for pregnant women, so you’ll have to validate your cuff first in a pregnant population because it may not be the same . . . that’s why I have to [ask patients to] bring all their cuffs in to calibrate, so [patients] always bring their home [blood pressure] cuffs in, and we calibrate them [at the clinic].

Provider 5

- Telemonitoring PHRPE may increase clinician workload: Some providers were concerned that BP self-monitoring for patients using telemonitoring would increase clinician workload. Reasons given included being alerted to abnormal BP readings during off-clinic hours, as well as patients requiring timely assessments, follow-up, and recommendations based on BP readings.

I mean in theory [telemonitoring] would be good, but who’s monitoring that is the problem . . . I think there’s enough information for us to deal with, and the expectation, I would just be a little bit hesitant to say that the expectation is for the physician to monitor the blood pressure . . . in terms of data that the patients are inserting into their app, because then it becomes a safety thing, because I’m not checking my phone all the time and I don’t think that should be the precedent.

Provider 1

#### Desired Features of a Telemonitoring Program

The desired features were as follows:

- Telemonitoring PHRPE should facilitate self-management for patients: Patients want to be able to easily transfer their BP results to a centralized location where it can be readily accessed by their provider. Both patients and providers commented on the ease they found using a diabetes monitoring system.

Because like me, I'm a diabetic, [and] my diabetes machine connects to my phone, so it's always synced there, so I see everything on my phone. And if I want to send it to my doctor, I just send a link to my doctor, and he can see every day what was my sugar.

Patient 2

Because when I think about what [patients] have to do, they have to create an email, like get an email and then write in all their readings. So if they could just have something that’s like a blood sugar monitor that monitored them and kept track of it, and put it into an app, I think [patients] would be onboard with that because it would be easier for them than having to keep track of all their numbers, then transfer it over into an email.

Provider 2

- Telemonitoring of PHRPE should facilitate decision making for clinicians: Providers suggested displaying BP results, medications, and symptoms to enable easy identification of abnormal values, trends, and the appropriate intervention.

In a busy clinic, it’s hard to sit and look at all the numbers, but imagine if patients plug a 2-week amount of blood pressure readings and then you get a graph. From that graph, you’ll know the highest and the lowest [numbers], you’ll know the trend, you’ll know the timing of day, and so you can, first of all, if things are worse at the end of the pregnancy, it might tell you to deliver. If you’re early on and showing you highest in the morning, then you might adjust nighttime [medication] dosing.

Provider 3

- Telemonitoring PHRPE should include evidence-based action prompts: Providers and patients highlighted the need for patient alerts and action prompts to be automatically generated from the patient data to ensure that patients seek medical attention, as needed.

I’m worried about [patients] throwing [blood pressure values] in [and] not knowing that, oh, like I’m waiting for somebody to get back to me, but meanwhile my blood pressures through the roof and I’m in danger, but because my app doesn’t tell me to do anything, I stay home.

Provider 1

I think that [getting feedback is] really important because . . . it empowers people to be more proactive about their health and what they should do and who they should reach out to.

Patient 3

Providers described the need for a DHI that can incorporate evidence-based protocols and standards for PHRPE to facilitate their decision making. Specifically, a provider discussed an online risk calculator developed by the Fetal Medicine Foundation based in the United Kingdom where maternal risk factors and biomarkers are input to calculate a patient’s risk for preeclampsia [20].

There is an online risk calculator that I personally use. It has been validated recently by a large study, published in [the] New England Medical Journal, so I actually use it to actually quantify the risk.

Provider 4

## Discussion

### Principal Findings

This paper presents the findings from a qualitative study aimed at determining the needs and challenges of monitoring PHRPE and the features required in a telemonitoring program to support the self-care and clinical management of PHRPE. Specifically, this study identifies participant perceptions on the benefits of, barriers to, and desired features of a telemonitoring program for PHRPE. Perceived benefits of telemonitoring for PHRPE included close monitoring of home BP measurements and appropriate interventions for abnormal BP readings; a tailored telemonitoring system for pregnant patients; and facilitation of self-management. Perceived barriers to telemonitoring for PHRPE included financial and personal barriers as well as the potential for increased clinician workload. Desired features of PHRPE included the facilitation of self-management for patients and decision making for clinicians, as well as the inclusion of evidence-based action prompts.

Patients and providers had similar opinions with respect to the features required in a telemonitoring program. For example, both patients and providers agreed that empowering patients is key to facilitating their self-management. Patients wanted feedback from a telemonitoring program so that they can feel empowered in their self-management of high BP during pregnancy. Patients looked to a telemonitoring program to educate themselves on what symptoms to identify, as well as the appropriate response when a symptom is identified. This viewpoint is similar to 2 recent studies published in the United Kingdom by Hinton et al [14]. One study described the acceptability and feasibility of self-monitoring BP during pregnancy from the women’s perspective [14]. The participants in this feasibility study reported feeling reassured and empowered by self-monitoring their BP, especially if they had a history of hypertension or preeclampsia [14]. Study participants felt that BP self-monitoring made them more knowledgeable of the risks of hypertension and preeclampsia in pregnancy [14]. The second study interviewed 147 obstetricians, community and hospital midwives, pharmacists, and trainee doctors to gain their perspective on the use of home BP monitoring during pregnancy [15]. In this study, providers acknowledged the potential for self-monitoring of BP to empower women in their health.

In our study, patients and providers had differing views on the perceived barriers to a telemonitoring program with respect to patient motivation. Patients who were less inclined to use technology for their health indicated that it would be difficult to incorporate telemonitoring into their routine. Moreover, depending on a patient’s situational context, such as the stage of pregnancy, incorporating telemonitoring into their life may become particularly challenging. For example, Hinton et al [14] noted that during the postpartum period, women found incorporating BP self-monitoring more challenging. Providers in our study noted that patient motivation is related to the patient's view of their disease and their lack of understanding related to its severity and impact on their health. A qualitative study by Davies et al [21] described primary care clinicians’ views on telemonitoring and highlighted that a lack of patient motivation is a barrier to their use. However, our study, with its small sample size as a limiting factor, demonstrated a highly motivated group in that all 7 patient participants measured their BP at home.

Both patients and providers referred to the ease with which telemonitoring systems or mobile apps for patients with diabetes send blood sugar readings to their providers. In fact, a summary of the recent studies on telemonitoring projects for patients with diabetes indicated favorable results, including improved control of blood glucose levels and a significant reduction in hemoglobin A^1c^, enhanced effects on comorbidities, increased quality of life for patients, and good uptake of the technology by patients [22].

A study published in 2019 by Band et al [16] incorporated theoretical modeling and a person-based approach to develop a logic model outlining the proposed mechanism of change for the Blood Pressure Self-Monitoring in Pregnancy (BUMP) program. This systematic approach to intervention development allowed for a deeper understanding and appreciation of the issues and experiences of pregnant patients with hypertensive disorders [16]. The logic model presents strategies, such as sending reminder prompts and cues to self-monitor and providing information about the benefits of self-monitoring, to address common barriers—barriers that were also reflected by the participants in our study to adopting self-monitoring during pregnancy [16].

Lastly, all providers were concerned about their clinical accountability with respect to a telemonitoring program for PHRPE. In fact, none of the providers had previously recommended the use of a telemonitoring program to their patients, stating the lack of availability of such telemonitoring systems and the lack of available guidelines for the telemonitoring of PHRPE. Similarly, Hinton et al [15] described the requirements identified by health care providers for telemonitoring programs to consider normal BP fluctuations throughout pregnancy and to ensure the accuracy of BP results in BP self-monitoring. Additionally, providers raised concerns about patients appropriately addressing abnormal BP [15]. These perspectives are also highlighted by a systematic review of the self-monitoring of BP in hypertension, which recommends the use of guidelines on how to interpret BP measurements taken at home, acceptable variances between home and clinic measurements, and how to direct patients on addressing concerning results [23].

### Recommendations on Features of the Telemonitoring Program

Both patient and provider participant groups expressed interest in the potential benefits of a telemonitoring program for PHRPE. The following features represent recommendations that could be used in the design and development of future programs:

- Assess patients’ technical literacy, motivation, and readiness to participate in a telemonitoring program preimplementation.
- Generate automatic alerts for patients when BP results are out of range, and instruct patients to either seek medical care or modify their self-care behaviors, depending on the BP value and trends, which would help address concerns related to clinician accountability.
- Develop alerts based on evidenced-based protocols and guidelines specific to PHRPE.
- Enable patients to add symptoms or other related information about their condition (eg, medications taken), in addition to BP readings, to better understand potential root causes for the symptoms.
- Send BP values automatically from the BP monitor to the telemonitoring system to avoid manual entry of data.
- Display patient data in a way that facilitates identification of trends and easy visualization of a patient’s health status by the provider.

### Recommendations on Frameworks for Program Implementation

Patient empowerment to facilitate self-management was a common theme discussed by both patient and provider participants. A framework that uses an empowerment-based approach to developing digital intervention tools for self-management has been described by Alpay et al [24]. This framework includes 6 components of patient empowerment (ie, communication, education and health literacy, information, self-care/support, decision aids, and contact with fellow patients) and may be beneficial in the implementation of a telemonitoring program for PHRPE [24]. This empowerment-based approach combined with the logic model, which was developed using a person-based approach proposed by Band et al [16], may provide direction on the design and implementation of a telemonitoring program for patients at high risk for hypertensive disorders of pregnancy.

### Limitations

Although the sample size was small and homogenous, with 7 patients and 5 health care providers being interviewed from a single health care institution, we felt that participants tended to repeat the same themes, which satisfied our requirement for data saturation. Additionally, study results cannot be generalizable to other women at risk for hypertension, since the study took place in a specialized renal clinic located in an urban setting.

### Conclusion

This study aimed to better understand the perceptions and needs of PHRPE and their health care providers with respect to telemonitoring. Through semistructured interviews with patients and providers, the benefits of, barriers to, and desired features of a telemonitoring program were identified. Patients and providers were hopeful about the benefits that such a program may provide in terms of self-care and clinical management of PHRPE. Perceived benefits included close monitoring of patients; tailored access to health care services, when needed; and a sense of empowerment for self-management. Perceived barriers to the telemonitoring of PHRPE included financial barriers as well as a potential increase in clinician workload. Desired features included the facilitation of self-management by patients, facilitation of decision making by providers, and provision of evidenced-based action prompts. Recommended features for a telemonitoring program for PHRPE were provided and were based on the perceived benefits, barriers, and desired features, as described by the patient and provider participants. Additionally, the use of theoretical frameworks in the design and implementation of a telemonitoring program for PHRPE, such as the empowerment-based approach for self-management and the person-based approach used to develop a logic model, were discussed as potential beneficial tools. The findings from this study validate the need for telemonitoring programs for PHRPE. The recommendations from this research may provide valuable insights into the development of future telemonitoring programs to improve self-care and the clinical management of PHRPE.

## Appendix

Multimedia Appendix 1 Interview guides.

## Abbreviations

BP: blood pressure
BUMP: Blood Pressure Self-Monitoring in Pregnancy
DHI: digital health intervention
PHRPE: patients at high risk for preeclampsia
