# Supplementary material for: Patients’ and Providers’ Perspectives on and Needs of Telemonitoring to Support Clinical Management and Self-care of People at High Risk for Preeclampsia: Qualitative Study
Source: JMIR Hum Factors. 2022 Feb 7;9(1):e32545. doi: 10.2196/32545 (PMC8861860; doi:10.2196/32545)
Supplement: Multimedia Appendix 1 [file humanfactors_v9i1e32545_app1.docx]

Healthcare Staff Interview Guide

Thank you for choosing to take part in this study. You can choose not to answer any question you do not want to answer and you may withdraw your participation at any point without any consequences or explanation.

I will now ask you some questions.

1. What is your current role in managing patients at-risk for pre-eclampsia?
   1. How many patients at-risk for pre-eclampsia do you see in your clinic in a year? Has this changed within the last 5 years?
   2. What are the demographics of these patients?
2. Can you tell me how patients at-risk for pre-eclampsia are managed at the clinic?
   1. What clinical markers are monitored during pregnancy or in the postpartum period?
   2. Is there a tool (paper-based, digital or other) to assist healthcare staff monitor these patients?
3. What education do you provide patients at-risk for pre-eclampsia during clinic visits?
   1. Do you recommend self-care behaviours like taking blood pressure self-measurements at home to these patients?
   2. Do you provide patients with educational material (brochures, pamphlets) or other resources (website, support groups)?
4. Are there barriers to supporting the self-care activities of patients who are at-risk for pre-eclampsia? Are there barriers to the clinical management of these patients?
5. Do you ever recommend the use of mobile health apps to patients at-risk for pre-eclampsia? If so, what app(s) do you recommend?
   1. Do you think a mobile health app can be used to support the self-care activities of patients at-risk for pre-eclampsia? Why or why not? How?
   2. Do you think a mobile health app can be used to clinically monitor patients at-risk for pre-eclampsia? Why or Why not? How?
   3. Do you think a mobile phone-based telemonitoring program would be useful in your clinic? Do you think it would work in your clinic?
   4. Do you see any barriers to implementing or using this kind of a program in your clinic?
6. Is there anything else you would like to add that we did not talk about?

Thank you for your time and participation in this study!

Patient Interview Guide

Thank you for choosing to take part in this study. You may choose not to answer any question you do not want to answer and you may withdraw your participation at any point without any consequences or explanation.

I will now ask you some questions.

1. How far along are you in your pregnancy?
2. Why were you referred to the Pregnancy and Kidney Disease Clinic?
3. How do you take care of yourself or manage your health (physical, mental, etc.) related to your pregnancy?
   1. Do you follow a specific diet or exercise program?
   2. How did you choose to take care of yourself in this way? Was it recommended to you by your healthcare provider? A friend or family member?
   3. Do you check your blood pressure at home? How? How often? What are some reasons you would check your blood pressure?
4. What do you think about taking your own blood pressure measurements at home (i.e., in between clinic visits)? Are there benefits/challenges to doing this?
   1. What would be the best method to share your blood pressure measurements with your healthcare provider? Manual entry or automatic transfer via Bluetooth?
5. Do you have someone (outside of clinic staff) who helps you manage your health related to your pregnancy, like a partner, friend, co-worker? If so, how do they help you?
6. Have you faced any challenges managing your health related to your pregnancy? If so, what kind of challenges have they been? If not, how have you avoided these challenges?
7. Have you ever used a mobile health app? If yes, which ones? If not, why not?
8. Do you think that mobile health apps can help you manage your health related to your pregnancy? Why or why not?
   1. What would this app be able to do?
   2. Can you picture yourself using this app?
9. Would it be useful to have a mobile phone-based telemonitoring program where your healthcare providers can monitor your condition at home?
10. Is there anything else you would like to add that we did not talk about?

Thank you for your time and participation in this study!
